# Municipal Wastewater Effluents as a Source of Listerial Pathogens in the Aquatic Milieu of the Eastern Cape Province of South Africa: A Concern of Public Health Importance

**DOI:** 10.3390/ijerph7052376

**Published:** 2010-05-12

**Authors:** Emmanuel E.O. Odjadjare, Larry C. Obi, Anthony I. Okoh

**Affiliations:** 1 Applied and Environmental Microbiology Research Group (AEMREG), Department of Biochemistry and Microbiology, University of Fort Hare, Private Bag X1314, Alice 5700, South Africa; E-Mail: eodjadjare@ufh.ac.za; 2 Deputy Vice-Chancellor office, Walter Sisulu University, Umthata, South Africa; E-Mail: obil@wsu.ac.za

**Keywords:** water quality, *Listeria* pathogens, health/environmental impact, receiving watershed

## Abstract

We evaluated the effluent quality of an urban wastewater treatment facility in South Africa and its impact on the receiving watershed for a period of 12 months. The prevalence and antimicrobial susceptibility of potential *Listeria* pathogens (*L. ivanovii* and *L. innocua*) and the physicochemical quality of the treated wastewater effluent was assessed, with a view to ascertain the potential health and environmental hazards of the discharged effluent. Total listerial density varied between 2.9 × 10^0^ and 1.2 × 10^5^ cfu/mL; free living *Listeria* species were more prevalent (84%), compared to *Listeria* species attached to planktons (59–75%). The treated effluent quality fell short of recommended standards for turbidity, dissolved oxygen, chemical oxygen demand, nitrite, phosphate and *Listeria* density; while pH, temperature, total dissolved solids and nitrate contents were compliant with target quality limits after treatment. The *Listeria* isolates (23) were sensitive to three (15%) of the 20 test antibiotics, and showed varying (4.5–91%) levels of resistance to 17 antibiotics. Of seven resistance gene markers assayed, only *sulII* genes were detected in five (22%) *Listeria* strains. The study demonstrates a potential negative impact of the wastewater effluent on the receiving environment and suggests a serious public health implication for those who depend on the receiving watershed for drinking and other purposes.

## 1. Introduction

*Listeria* is an emerging pathogen commonly associated with foodborne infections. Although seven species are recognized namely *L. monocytogenes*, *L. ivanovii*, *L. innocua*, *L. seeligeri*, *L. welshimeri*, *L. grayii* and *L. murrayi*, only two (*L. monocytogenes* and *L. ivanovii*) are pathogenic; the former is responsible for disease in both humans and animals, while the latter causes diseases mostly in ruminants but also in other animals [1,2]. There are reports, however, of *L. seeligeri* and *L. ivanovii* causing illnesses in humans [3,4], and *L. innocua* is occasionally associated with encephalitis in ruminants [5]. Other species are generally regarded as non-pathogenic [2].

The bacterium has been implicated in several foodborne outbreaks in the developed world [6,7] with little information on the existence of the pathogen in developing countries [6]. Although food is reported to be the major route of transmission of the pathogen, previous studies [8–13] indicated that *Listeria* is capable of surviving conventional wastewater treatment process even after disinfection; thus suggesting that wastewater may be significant in the epidemiology of the pathogen. This has serious public health implications for developing countries such as South Africa where a larger percentage of the population depend on surface water bodies that may be negatively impacted by untreated or inadequately treated wastewater for drinking and other purposes [14–16]. The existence of bacteria as free-living or attached cells was previously observed [17–19] to influence their capacity to resist disinfection and enhance resistance to antimicrobial therapy. Listerial resistance to antimicrobial therapy was also reported [20,21] to be mediated by certain resistance genes that encodes proteins which function in ways that inhibit or reduce the effects of antimicrobials on the pathogen.

*Listeria* infections have the highest (up to 50%) mortality rate amongst foodborne pathogens [6], making the South African public particularly vulnerable in the event of an outbreak due to the high HIV/AIDS prevalence level and rate of drug and alcohol abuse in the country [22]. The potential severity of listeriosis outbreak on the public health notwithstanding, there is dearth of information on the prevalence of this pathogen in South Africa. More worrisome is the fact that globally, *Listeria* is not considered a waterborne pathogen in spite of reports in the literature [8–13,21] suggesting that the pathogen is well established in the water supply chain.

The etiology of many waterborne outbreaks in South Africa is not known [23]; this may be due to the religious focus on traditional waterborne pathogens by investigators. The dire need to preserve the public health however calls for the investigation of emerging waterborne pathogens that were hitherto not investigated or overlooked notwithstanding their potentials to survive and distribute within the water supply chain. The current study was therefore carried out to investigate the effluent quality (*Listeria* pathogens and physicochemical) of a typical urban wastewater treatment facility in South Africa and its impact on the receiving watershed; with emphasis on the potential public health and environmental hazards associated with the use of such waters.

## 2. Materials and Methods

### 2.1. Description of Sampling Site

**Figure 1.**
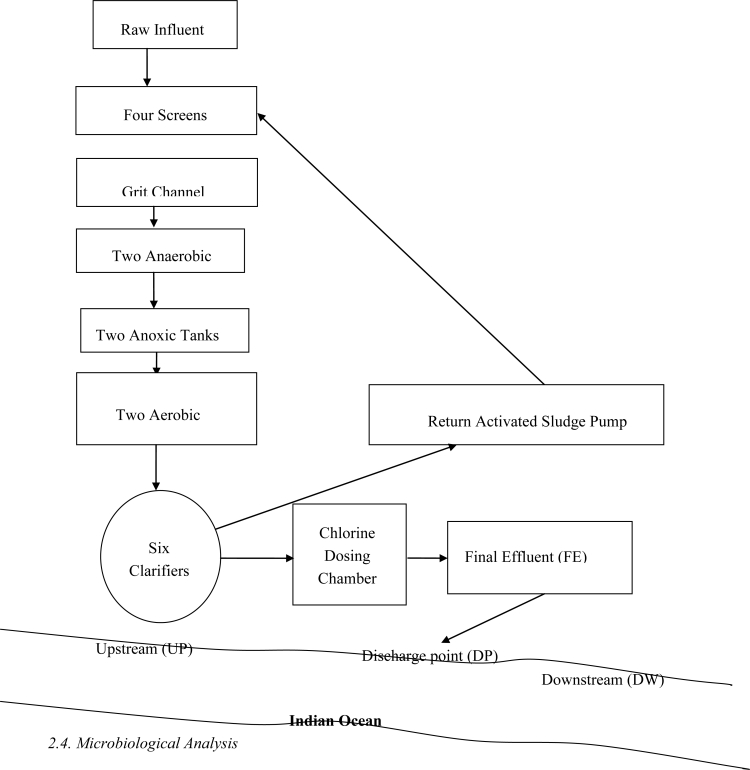
Schematic representation of the study area.

The wastewater treatment plant (Figure 1) is located in East London, a large and highly populated urban community in the Eastern Cape Province of South Africa, with the geographical coordinates: 32.97°S and 27.87°E. The plant receives municipal domestic sewage and a heavy industrial effluent and comprise of four screens, a grit channel, two anaerobic and two anoxic tanks and two aerobic tanks (each equipped with three vertically mounted mechanical aerators). The plant has six sedimentation tanks with the return activated sludge (RAS) pumped from the bottom of the clarifiers via the screens with raw sewage to the aeration tanks. Chlorine contact is carried out by means of a water pressure operated, wall mounted, gas chlorinator in a baffled reinforced concrete contact tank and the final effluent is discharged into the Indian Ocean. The average daily inflow of raw sewage during the study period was 32,000 m^3^/day, while the plant has a built in capacity of 40,000 m^3^/day.

### 2.2. Sample Collection

Wastewater samples were collected on a monthly basis from the final effluent (FE), discharge point (DP), five hundred meters (500 m) upstream (UP) and five hundred meters (500 m) downstream (DW) of the discharge point between August 2007 and July 2008. Aqueous effluent samples were collected in duplicates in sterile one liter Nalgene bottles and transported in cooler boxes containing ice packs to the Applied and Environmental Microbiology Research Group (AEMREG) laboratory at the University of Fort Hare, Alice, South Africa for analyses. Sample bottles for the final effluents contained 0.1% sodium thiosulphate (3% solution) to neutralize the effect of the chlorine residual on the microflora. Processing of samples was done within 6 hours of sample collection.

### 2.3. Sample Processing

Samples were processed according to the descriptions of Maugeri *et al.* [24] with modifications. Briefly, samples (one liter in duplicates) were filtered in the laboratory through 180-, 60- and 20-μm pore size nylon nets (Millipore Corp., Ireland) respectively; the water that flowed through the 20-μm pore size nylon nets was collected in clean sterile containers for planktonic (free-living) *Listeria* cells analyses. To obtain a final volume corresponding to 40× of the original sample, trapped planktons on the nets and adhering bacteria were resuspended in 25 mL of sterile phosphate-buffered saline (PBS). To detach adhering bacteria from the planktons, 12.5 g of sterile 0.1 mm glass beads (Biospec Products Inc., Bartlesville, OK 74005, USA) was weighed into the bacteria-plankton suspension, vortexed at high speed for 30 s and centrifuged at 3,000× g for 10 min at ambient temperature using the Beckman Model TJ-6 centrifuge. The glass beads were allowed to settle to the bottom of the centrifuge tube and the supernatant was used for plankton-associated *Listeria* analyses. Henceforth in this paper, plankton of sizes ≥ 180 μm, ≥ 60 μm ≤ 180 μm, and ≥ 20 μm ≤ 60 μm, shall simply be represented as 180 μm, 60 μm and 20 μm, respectively.

### 2.4. Microbiological Analysis

The isolation of *Listeria* species were done according to the description of Hitchins [25] with modifications. Briefly, aliquots of samples containing free-living and plankton-associated bacteria were directly inoculated onto *Listeria* chromogenic agar (LCA agar) (Pronadisa^®^ Madrid, Spain) following standard spread plate technique and incubated for 24–48 h at 35 °C. Typical *Listeria* colonies appear blue-green on LCA agar plates while pathogenic *Listeria* species (*Listeria monocytogenes* and *L. ivanovii*) were surrounded by an opaque halo in addition to their blue-green color. Total *Listeria* counts were recorded and presumptive *Listeria* pathogens were isolated from the treated (chlorinated) effluent samples, purified and stored on nutrient agar slants at 4 °C for further analyses. The presumptive *Listeria* pathogens were further confirmed by standard cultural characteristics and biochemical reactions [25] and using the API *Listeria* kits (10 300, bioMerieux, South Africa). *Listeria monocytogenes* (ATCC 19115) and *Staphylococcus aureus* (ATCC 25923) were used as positive and negative controls, respectively.

### 2.5. Physicochemical Analyses

All field meters and equipment were checked and appropriately calibrated according to the manufacturers’ instructions. pH, temperature, total dissolve solid (TDS), and dissolved oxygen (DO), were all determined on site using the multi-parameter ion specific meter (Hanna-BDH laboratory supplies). Turbidity and the concentrations of free chlorine residual in the final effluent samples were also determined on site using a microprocessor turbidity meter (HACH Company, model 2100P) and an ion-specific meter (Hanna Instruments, HI 93711) respectively. The concentrations of orthophosphate as P (PO_4_), nitrate (NO_3_), nitrite (NO_2_), and chemical oxygen demand (COD) were determined in the laboratory by the standard photometric method [26] using the spectroquant NOVA 60 photometer (Merck Pty Ltd). Samples for COD analyses were digested with a thermoreactor model TR 300 (Merck Pty Ltd) prior to analysis using the spectroquant NOVA 60 photometer.

### 2.6. Antimicrobial Agents

Twenty antibiotics commonly used as therapy in human and veterinary listeriosis were employed in the antibiogram assay. The paper disks containing the antibiotics were obtained from Mast Diagnostics (Merseyside, United Kingdom) and includes: Amikacin (30 μg), Ciprofloxacin (5 μg), Aztreonam (30 μg), Linezolid (30 μg), Chloramphenicol (30 μg), Imipenem (10 μg), Ceftriaxone (30 μg), Meropenem (10 μg), Cephalothin (30 μg), Ertapenem (10 μg), Erythromycin (15 μg), Gatifloxacin (5 μg), Gentamicin (10 μg), Moxifloxacin (5 μg), Ampicillin (25 μg), Streptomycin (25 μg), Penicillin G (10 μg), Tetracycline (30 μg), Trimethoprim (5 μg), and Sulphamethoxazole (25 μg).

### 2.7. Antibiotic Susceptibility Test

The antibiotic susceptibility test was performed and interpreted based on the disk diffusion method as described by the Clinical and Laboratory Standard Institute [27], using Mueller Hinton agar plates (Biolab, Merck, South Africa). The inhibition zone diameters (IZD) were interpreted according to CLSI standards for staphylococci due to lack of specific standards for *Listeria* species [28]. Interpretative standard for Linezolid was still under investigation for staphylococci at the time of this report, thus standard for *Enterococcus* species was applied for this antimicrobial agent.

### 2.8. Bacterial DNA Extraction and Amplification of Antimicrobial Resistance Genes

DNA was isolated from pure cultures of the selected *Listeria* strains by the boiling method as described elsewhere [29]. Based on the *in vitro* antimicrobial susceptibility profile of the *Listeria* isolates, seven antimicrobial resistance genes including those encoding penicillin binding protein (*penA*); dihydropteroate synthetase type I (*sulI*); dihydropteroate synthetase type II (*sulII*); adenine methylase (*ermA*); erythromycin resistance methylase (*ermB*); erythromycin esterase type II (*ereB*); and β-lactamase-ampicillin resistance gene (*ampC*); were selected for screening. Oligonucleotide sequences and predicted amplicon sizes for the different antimicrobial resistance genes are listed in Table 1. Presence of antimicrobial resistance genes in the *Listeria* species were all determined by PCR technique according to the description of Srinivasan *et al.* [21].

### 2.9. Statistical Analyses

Calculation of means and standard deviations were performed using Microsoft Excel Office 2007 version. Correlations (paired T-test) and test of significance (one-way ANOVA) were performed using SPSS 17.0 version for Windows program (SPSS, Inc.). All tests of significance and correlations were considered statistically significant at *P* values of < 0.05 or < 0.01.

## 3. Results

### 3.1. Abundance of Listeria

Total *Listeria* counts ranged from 2.9 × 10^0^ to 1.2 × 10^5^ cfu/mL (Table 2). The lowest count was observed during summer in the month of November 2007 at DW while the highest count was observed at the DP, also in the summer month of December 2007. Abundance of free-living *Listeria* species varied between 0 and 2.4×10^3^ cfu/mL, with the highest count recorded at FE and DW in April 2008. *Listeria* species associated with plankton of sizes 180 μm, 60 μm, and 20 μm, were observed at population densities of 0 to 1.95 × 10^3^ cfu/mL, 0 to 1.8 × 10^2^ cfu/mL and 0 to 1.15 × 10^5^ cfu/mL, respectively. The highest counts for the plankton-associated *Listeria* species were all observed at the DP in December 2007, June 2008 and December 2007 for 180 μm, 60 μm, and 20 μm categories, respectively. Listerial abundance did not vary significantly with season either as free-living or plankton-associated entities. The population of free-living *Listeria* species in the FE samples varied significantly (*P* < 0.05) with those of large (180 μm) and medium sized (60 μm) planktons but not with small (20 μm) planktons. *Listeria* density did not vary significantly with the size of the planktons to which they attach at DP and DW. There was, however, significant difference (*P* < 0.05) in listerial density between free-living *Listeria* populations and plankton-attached species of all categories at the UP sampling site.

There was significant (*P <* 0.01) positive correlation between *Listeria* populations attached to large (180 μm) planktons and those attached to small (20 μm) planktons. Significant correlation was, however, not observed for other treatments with respect to listerio-plankton association.

Table 2 also shows the prevalence of *Listeria* during this study. *Listeria* species were isolated throughout the year from the treated effluents and the receiving watershed. Thirty-seven (84%) of all 44 samples (in duplicate) were positive for free-living *Listeria* species. Free-living *Listeria* species were isolated all year round except in DW (summer and early winter: May, 2008) and in UP (December 2007; May and June 2008). Seventy-five percent of all samples were positive for *Listeria* species associated with large (180 μm) plankton. Of these, *Listeria* was isolated from FE (11 samples), DP (nine samples), DW (seven samples) and UP (six samples). Twenty-six (59%) of all 44 samples were positive for *Listeria* species associated with medium-sized (60 μm) planktons, which were isolated from FE (10 samples), DP (eight samples), DW (three samples) and UP (five samples). *Listeria* species associated with small (20 μm) planktons were isolated in 30 (68%) of the 44 samples. FE samples were positive for this *Listeria* species in 10 samples, DP in nine samples, DW in five samples and UP in six samples.

### 3.2. Physicochemical Analyses

**Figure 2.**
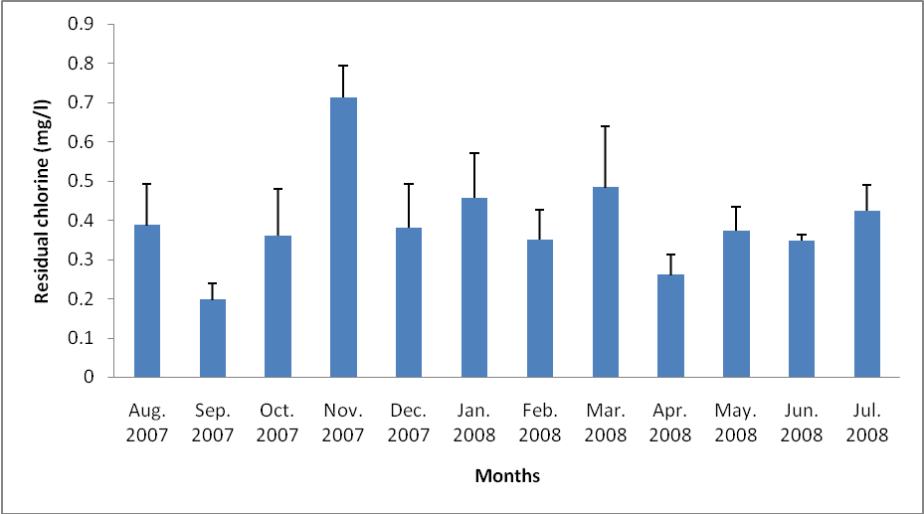
Chlorine residual regime of the treated effluents during the 12 month study period.

**Figure 3.**
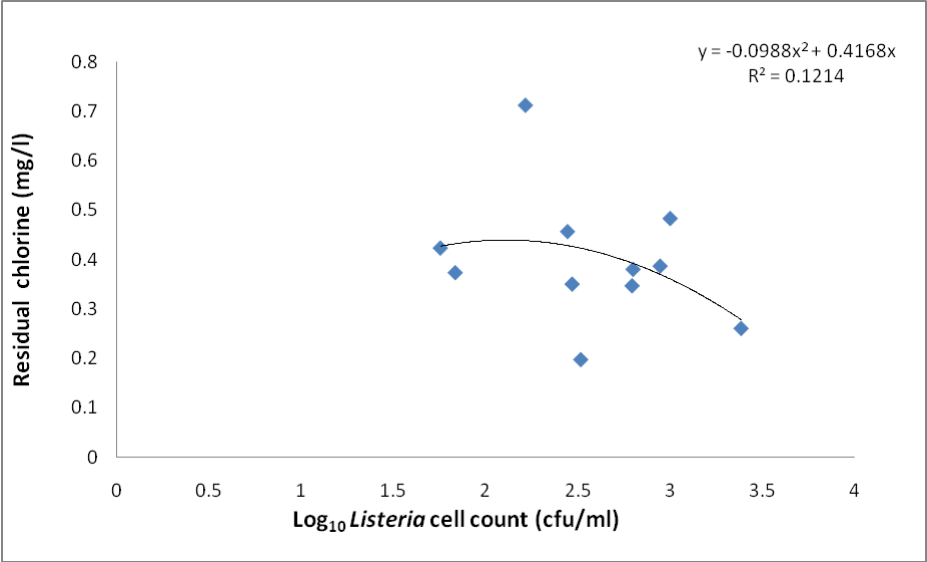
Scatter plot of the relationship between listerial density (total *Listeria* count) and chlorine residual. Total listerial density was not determined for the final effluent in the month of October; hence the listerial density for that month is not reflected in the figure.

Table 3 shows the range and annual mean values of some wastewater quality parameters before and after treatment of the wastewater under study. Significant differences was observed between raw sewage and treated effluent in terms of turbidity, DO, and PO_4_ (*P <* 0.01) and for nitrate (*P <* 0.05). There was, however, no significant difference between treated and untreated wastewater for pH, temperature, TDS, COD, and NO_2_. Figure 2 shows the free chlorine residual (CR) of the final effluents during the 12 month study period. Chlorine residual ranged between 0.197 mg/L (September, 2007) and 0.71 mg/L (November, 2007). The relationship between residual chlorine and total *Listeria* count did not follow any defined trend (Figure 3).

### 3.4. Antibiogram and Resistance Gene Detection

Fifty-one presumptive *Listeria* pathogens were isolated from the final effluents following their morphological characteristics on LCA plates. Of the 51 isolates, 27 (53%) were confirmed to be *L. ivanovii*; 1 (2%) was *L. innocua* and the identity of the remaining 23 (45%) isolates were indeterminate by API test. Twenty-three (22 *L. ivanovii* and 1 *L. innocua*) of the 28 confirmed *Listeria* isolates were tested for phenotypic antibiotic susceptibility and the result is shown in Table 4. All 23 *Listeria* species were sensitive to three (15%) of the 20 test antibiotics including amikacin (aminoglycosides), meropenem, and ertapenem (carbapenems). Eight (35%) of the 23 *Listeria* isolates were moderately sensitive to moxifloxacin, cephalothin, gatifloxacin, ciprofloxacin and ceftriaxone; three strains showed moderate sensitivity to moxifloxacin, two to gatifloxacin, while the other three were each moderately sensitive to cephalothin, ciprofloxacin, and ceftriaxone. The test isolates showed resistance to 17 (85%) of the 20 antibiotics at percentages ranging from 4.5% to 91% (Table 4). Multiple antibiotic resistances was observed in 22 (95.7%) of the isolates in combinations ranging from four to 10 antibiotics; while one isolate showed resistance to a single antibiotic (aztreonam) (Table 5). Of the seven antimicrobial genes assayed in this study, only *sulII* genes were detected in five (22%) strains of *Listeria ivanovii* (Table 6).

## 4. Discussion

The relative abundance of free-living *Listeria* species found during this study and across all sampled sites is consistent with reports elsewhere [13,35]. There are no recommended standards specific for *Listeria* pathogens in water and wastewater samples in South Africa for obvious reasons; thus the fecal coliforms standard (0 cfu/100 ml) for domestic water uses [30] was applied in this report. Based on this standard, the water quality across the studied water system and throughout the year (Table 2) fell short of acceptable target limits for domestic applications, thus disqualifying the waters for use in drinking and other domestic purposes. *Listeria* abundance did not vary significantly with season, either as free-living or plankton-associated species, consistent with the observation of Murrel *et al.* [36], but contrary to our previous report [13]. The significant positive correlation observed between *Listeria* species attached to large (180 μm) planktons and those attached to small (20 μm) planktons suggests that the two groups of *Listeria* species may occupy the same niche in the ecosystem; this is contrary to our previous report [13], where *Listeria* species attached to large (180 μm) planktons negatively correlated with those attached to small (20 μm) planktons. The lack of significant correlations between and among other treatments in this study suggests that free-living *Listeria* species and *Listeria* species attached to medium-sized (60 μm) planktons occupy separate niches in the water system, different from those occupied by *Listeria* species attached to large (180 μm) and small (20 μm) planktons. The observation is consistent with those of Maugeri *et al.* [24] who reported lack of significant correlation between free-living bacteria and plankton associated bacterial populations in a marine coastal zone in Italy. However, another study [37] reported a negative correlation between planktonic *Vibrio* cells and sessile populations.

*Listeria* species were isolated from all sampled sites and throughout the year during this study, suggesting a 100% prevalence of the pathogen in the water system. Consistent with observations in a previous study [13], free-living *Listeria* species were most prevalent (84%) both in treated effluent and the receiving watershed; followed by *Listeria* cells associated with planktons of sizes 180 μm (75%), 20 μm (68%), and 60 μm (59%), respectively. Corroborating this observation, Maugeri *et al.* [24] reported higher prevalence for free-living bacteria compared to their plankton-associated counterparts. *Listeria* species were generally more prevalent in the treated effluents (FE), both as free-living and/or plankton-associated cells, compared to the receiving watershed (Table 2). The observation could be as a result of higher nutrient levels in the wastewater effluents compared to the receiving watershed, in agreement with previous reports [10,11,38]. Consistent with the observation of this study, high prevalence of *Listeria* species has been reported in water systems impacted by wastewater effluents in Iraq [8,9], Poland [10] France [11], the United Kingdom [12] and rural South Africa [13]. Watkins and Sleath [12] reported 100% prevalence of *Listeria* species in sewage, river water, and trade effluent at densities (7.0 × 10^2^ to >1.8 × 10^4^ Most Probable Number (MPN)/mL), slightly higher than those observed in this study. The sewage effluent reported by Watkins and colleague, however, only underwent primary treatment unlike ours that was disinfected by chlorination, which could account for the differences. Al-Ghazali and Al-Azawi [8,9] also reported 100% prevalence in treated wastewater effluent in Iraq but at lower densities of <3 to 28 MPN/mL, and Paillard *et al.* [11] reported 84.4% prevalence of *Listeria* species in treated wastewater in France at densities ranging from <0.3 to 21 MPN/ml, while Odjadjare and Okoh [13] recorded 100% prevalence in a rural water system in South Africa at densities ranging from 1.0 × 10^1^ to 1.1 × 10^4^ cfu/mL. Contrary to the observation of this study, lower prevalence has been reported for *Listeria* species in a variety of surface water systems. Frances *et al.* [39] reported the isolation of *Listeria* species from 21% of freshwater samples collected from sites in Cheshire and North Wales; while Lyautey *et al.* [40] reported 64% for surface waters of the South Nation River Watershed in Ontario, Canada. These observations were consistent with expectations for surface waters that were not impacted by wastewater effluent in agreement with a report elsewhere [38].

The significant variation observed between raw and treated sewage for most physicochemical parameters (Table 3) is an indication that the wastewater treatment process remarkably improved the quality of the raw wastewater influent. However, despite the improvement on raw sewage quality, the treated effluent did not measure up to the desired target quality for turbidity, DO, COD, and NO_2_ with respect to domestic applications [30] and PO_4_ with reference to preserving the integrity of the aquatic ecosystem [34]. This suggests that the wastewater effluent has a potential negative impact on the environment and public health. The effluent quality was, however, acceptable in terms of pH, temperature, TDS, and NO_3_ (Table 3).

The chlorine residual (Figure 2) generally fell within acceptable target limits (0.3–0.6 mg/L) for domestic water at the point of use [41], except in September and November 2007, and indicates that the water is safe for domestic applications with reference to chlorine residual. The scatter plot (Figure 3) indicates that the relationship between chlorine residual and listerial density did not follow any particular trend. This observation suggests that factors other than chlorine disinfection affected the abundance of *Listeria* species during this study; some of these factors may also be responsible for the inability of chlorine to adequately eliminate the pathogens from the wastewater even at relatively high doses. LeChevallier *et al.* [42] observed attachment of bacteria to planktons and/or other suspended particles as a factor which enhanced resistance of bacteria to chlorine disinfection while Obi *et al.* [41] reported other factors to include contact time, temperature, and pH. This suggests that turbidity (which is a measure of suspended particles including planktons) could be a factor in the ineffectiveness of chlorine disinfection during this study; turbidity fell short of recommended target limits throughout the study (Table 3). Attachment of *Listeria* species to plankton may, however, not be a significant factor in the bacterial survival of chlorine disinfection in this study, as free-living *Listeria* species were more abundant compared to their plankton attached counterparts even after chlorine disinfection in agreement with the observation of our study elsewhere [13]. The reason for this observation is not clear.

Previous studies on the antimicrobial susceptibility profiles of *Listeria* species focused mainly on clinical and/or food isolates with little information in the literature on antibiotic susceptibility profiles for *Listeria* strains isolated from chlorinated municipal wastewater effluent. All 23 *Listeria* species tested in this study were sensitive to three (15%) of the 20 test antibiotics including amikacin (aminoglycosides), meropenem, and ertapenem (carbapenems) (Table 4); suggesting that these antibiotics may be the best therapy in the event of listeriosis outbreak in South Africa. Consistent with the observation of this study, Hansen *et al.* [43] reported complete sensitivity of 106 *Listeria* species isolated from humans to meropenem, while Safdar and Armstrong [44] observed 100% sensitivity to amikacin and Odjadjare and Okoh [13] reported complete sensitivity to the three antibiotics by all 14 *Listeria* species isolated from chlorinated wastewater effluent in a previous study.

*Listeria* strains in this study showed resistance to at least one of 17 antibiotics at percentages ranging from 4.5%–91% (Table 4), and particularly high levels for penicillin G (91%), ampicillin (87%), erythromycin (83%), and sulphamethoxazole (65%). Contrary to the observation of this study, *Listeria* species were generally reported to be susceptible to penicillin G [45], ampicillin [46], erythromycin [28,44], and sulphamethoxazole [13,43]. Conversely, considerable resistance has been reported in the literature for *Listeria* species against the penicillins (penicillin G and ampicillin) [21], erythromycin [13,47], and sulphamethoxazole [46]. The high resistance observed for penicillin G, ampicillin and sulphamethoxazole could be of serious public health concern as penicillin G and ampicillin are reported to be the antibiotics of choice in the treatment of listeriosis [28,43]; while sulphamethoxazole, usually in combination with trimethoprim, is considered second choice therapy, especially for patients who are allergic to the penicillins [46]. The observation generally indicated that municipal wastewater effluent could be a significant source of highly resistant strains of *Listeria* pathogens in the South African aquatic milieu.

The physicochemical quality of the wastewater effluent may be a factor in the level of resistance observed in this study, as it is widely reported [48–50] that conventional wastewater treatment plants lack the capacity to effectively remove antibiotics and a number of other chemicals from wastewater, thereby increasing the chances of bacterial pathogens resident in such wastewater effluent to develop resistance to common antibiotics due to selective pressure. Although we did not attempt to assay for residual antibiotics in the treated effluents in the course of this study, lack of capacity to remove some chemicals from the wastewater during the treatment process is evident in Table 3. The table shows that the treated effluent fell short of recommended standard quality for critical parameters such as turbidity, DO, COD, NO_2_, and PO_4_ and suggests a possible influence on the listerial resistance.

Twenty-two (95.7%) of the 23 test isolates in this study showed multiple antibiotic resistance in combinations ranging from four to 10 antibiotics (Table 5). Similar observations have been reported elsewhere [13,21]. On the contrary Conter *et al.* [28] reported more resistance to single antibiotics than multiple resistance amongst 120 *Listeria* isolates tested against 19 antibiotics; while Arslan and Ozdemir [51] reported resistance to single antibiotics with no record of multiple antibiotic resistance amongst 47 strains of *Listeria* species isolated from white cheese and tested against 13 antibiotics. Multiple drug resistance in *Listeria* species have been attributed to antimicrobial selective pressure and gene transfer mechanism between and among *Listeria* species and close relatives of the bacteria such as *Enterococcus*, *Streptococcus* and *Staphylococcus* species [44]. Donlan and Costerton [52] also reported the acquisition of inherent resistance to antimicrobial agents due to bacterial attachment to surfaces; suggesting that attachment to plankton at one point or the other may have enhanced the multiple resistances of our listerial strains to several test antibiotics.

Although the penicillins (penicillin G and ampicillin) and erythromycin showed the highest phenotypic resistance during this study, the genes responsible for resistance to these antibiotics were not detected in our *Listeria* isolates (Table 6). In a similar report, Srinivasan *et al.* [21] observed high level (92%) of phenotypic resistance to ampicillin but failed to detect the genes responsible for ampicillin resistance in all of their 38 *Listeria* isolates. Consistent with the observation of this study, Davis and Jackson [20] could not detect *penA* genes (responsible for penicillin resistance) in *Listeria* strains isolated from various sources; while Srinivasan *et al.* [21] reported their inability to detect genes responsible for erythromycin resistance in 38 *Listeria* isolates from dairy farms in spite of observed phenotypic resistance to the antibiotic. Contrary to the observation of this study, Srinivasan *et al.* [21] reported the detection of *penA* genes in 37% of their *Listeria* isolates while Roberts *et al.* [53] reported the detection of erythromycin resistance genes in *Listeria* species isolated from food samples. To the best of our knowledge, this is the first report on the detection of dihydropteroate synthetase type II (*sulII*) resistance gene markers in *Listeria* species (Table 6). Previous attempt by other workers [20,21] did not detect the genes in *Listeria* species. The percentage of *Listeria* isolates that harbored this gene was, however, relatively small (22%) compared to the high (65%) level of phenotypic resistance observed for the antibiotic (sulphametoxazole) in this study. The observations generally suggests that the presence of antimicrobial resistance genes in bacterial isolates do not always correlate with phenotypic antibiotic resistance, and indicates that other mechanisms such as decreased outer membrane permeability, activation of efflux pump, or mutation in a ribosomal protein may have contributed to the antimicrobial resistance phenotypes observed in this study [21].

## 5. Conclusions

The current study demonstrated that the activated sludge treatment process was ineffective in removing *Listeria* pathogens and other contaminants from the municipal wastewater prior to discharge into the receiving watershed; thereby posing serious threat to the integrity of the receiving environment and its ability to support life; as well as endangering the public health of the people who depend on this all important water resource for drinking and other purposes. Therefore, it is imperative that the relevant monitoring agencies take proactive steps aimed at curtailing an impending listeriosis outbreak in South Africa in the interest of the public health.

## Figures and Tables

**Table 1.** Primers used for resistance genes detection in the *Listeria* isolates from chlorinated waste water effluents.

Gene	Primer	Nucleotide sequence	Amplicon size	Reference
*penA*	PenA-F	ATCGAACAGGCGACGATGTC	500	[21]
	PenA-R	GATTAAGACGGTGTTTTACGG		
*ampC*	AmpC-F	TTCTATCAAMACTGGCARCC	550	”
	AmpC-R	CCYTTTTATGTACCCAYGA		
*ermB*	ErmB-F	GAAAAGGTACTCAACCAAATA	639	”
	ErmB-R	AGTAACGGTACTTAAATTGTTTAC		
*ereA*	EreA-F	AACACCCTGAACCCAAGGGACG	420	”
	EreA-R	CTTCACATCCGGATTCGCTCGA		
*ereB*	EreB-F	AGAAATGGAGGTTCATACTTACCA	546	”
	EreB-R	CATATAATCATCACCAATGGCA		
*su1I*	Su1I-F	GTGACGGTGTTCGGCATTCT	779	”
	Su1I-R	TCCGAGAAGGTGATTGCGCT		
*su1II*	Su1II-F	CGGCATCGTCAACATAACCT	721	”
	Su1II-R	TGTGCGGATGAAGTCAGCTC		

**Table 2.** Population density and distribution of the *Listeria* species in the treated effluents and its receiving watershed.

Net Sampling pore Sites sizes					***Listeria*density (cfu/mL)**								
		Spring			Summer			Autumn			Winter		

		Aug. 2007	Sep. 2007	Oct. 2007	Nov. 2007	Dec. 2007	Jan. 2008	Feb. 2008	Mar. 2008	Apr. 2008	May 2008	Jun. 2008	Jul. 2008
FE	180 μm	1.5×10^0^	3.5×10^0^	ND	4.0×10^0^	8.6×10^1^	2.5× 10^1^	7.6×10^0^	3.5×10^1^	1.1×10^1^	2.7×10^1^	4.3×10^1^	1.8×0^1^
	60 μm	2.9×10^0^	2.4×10^0^	ND	0.0	1.0×10^1^	1.6 ×10^1^	3.0×10^0^	1.4×10^1^	8.1×10^0^	1.0×10^1^	3.8×10^1^	1.2×10^1^
	20 μm	6.3×10^2^	7.1×10^0^	ND	0.0	3.0×10^2^	1.2×10^1^	9.3×10^0^	3.9×10^0^	9.4×10^0^	1.2×10^1^	9.3×10^1^	1.1×10^0^
	Free	2.6×10^2^	3.0 ×10^2^	ND	1.6×10^2^	2.4× 10^2^	2.3× 10^2^	2.8×10^2^	9.5×10^2^	2.4×10^3^	2.0×10^1^	4.5×10^2^	2.5×10^1^
	**Total**	**8.8×10^2^**	**3.3×10^2^**	**ND**	**1.7×10^2^**	**6.3×10^2^**	**2.8×10^2^**	**2.95×10^2^**	**1.0×10^3^**	**2.4×10^3^**	**6.9×10^1^**	**6.2×10^2^**	**5.7×10^1^**
DP	180 μm	3.9×10^0^	2.1×10^0^	ND	3.0×10^0^	1.95×10^3^	9.9×10^0^	1.5×10^0^	2.1×10^1^	0.0	1.0×10^1^	1.8×10^2^	0.0
	60 μm	3.5×10^0^	0.0	ND	0.0	1.9×10^1^	2.2×10^1^	3.8×10^0^	3.5×10^0^	7.6×10^0^	7.0×10^0^	1.8×10^2^	0.0
	20 μm	2.8×10^0^	1.1×10^0^	ND	0.0	1.2×10^5^	6.3×10^0^	6.1×10^0^	4.7×10^1^	6.7×10^1^	1.6×10^1^	6.9×10^1^	0.0
	Free	5.7×10^2^	2.1×10^2^	ND	1.5×10^1^	4.0×10^2^	8.0×10^1^	2.1×10^2^	3.4×10^2^	3.5×10^1^	1.5×10^2^	8.5×10^1^	5.0×10^0^
	**Total**	**5.8×10^2^**	**2.1×10^2^**	**ND**	**1.98×10^1^**	**1.2×10^5^**	**1.2×10^2^**	**2.2×10^2^**	**4.1×10^2^**	**1.1×10^2^**	**1.8×10^2^**	**5.1×10^2^**	**5.0×10^0^**
DW	180 μm	0.0	1.1×10^0^	ND	2.9×10^0^	0.0	2.1×10^1^	1.1×10^0^	2.9×10^0^	0.0	4.3×10^0^	2.6×10^1^	0.0
	60 μm	0.0	0.0	ND	0.0	0.0	1.5×10^1^	0.0	0.0	0.0	6.9×10^0^	3.0×10^1^	0.0
	20 μm	0.0	0.0	ND	0.0	0.0	1.2×10^1^	1.6×10^0^	9.6×10^0^	0.0	1.96×10^1^	1.8×10^1^	0.0
	Free	3.5×10^1^	3.5×10^1^	ND	0.0	0.0	0.0	5.0×10^1^	1.6×10^2^	2.4×10^3^	0.0	1.5×10^1^	5.0×10^0^
	**Total**	**3.5×10^1^**	**3.6×10^1^**	**ND**	**2.9×10^0^**	**0.0**	**4.8×10^1^**	**7.8×10^0^**	**1.7×10^2^**	**2.4×10^3^**	**3.1×10^1^**	**8.9×10^1^**	**5.0×10^0^**
UP	180 μm	0.0	0.0	ND	3.5×10^0^	0.0	2.5×10^1^	1.0×10^0^	4.4×10^0^	0.0	4.3×10^0^	9.9×10^0^	0.0
	60 μm	0.0	0.0	ND	0.0	0.0	8.9×10^0^	2.0×10^0^	1.1×10^0^	0.0	2.7×10^1^	2.4×10^1^	0.0
	20 μm	0.0	0.0	ND	3.6×10^3^	0.0	7.6×10^0^	1.5×10^0^	2.4×10^0^	0.0	1.7×10^1^	3.1×10^1^	0.0
	Free	1.5×10^1^	5.0×10^0^	ND	1.2×10^2^	0.0	3.5×10^1^	1.0×10^1^	1.3×10^2^	9.0×10^1^	0.0	0.0	5.0×10^0^
	**Total**	**1.5×10^1^**	**5.0×10^0^**	**ND**	**1.2×10^2^**	**0.0**	**7.6×10^1^**	**1.5×10^1^**	**1.4×10^2^**	**9.0×10^1^**	**4.8×10^1^**	**6.5×10^1^**	**5.0×10^0^**

Legend*: FE* = treated final effluent, *DP* = discharge point, *DW* = 500 m downstream discharge point, *UP* = 500 m upstream discharge point; *ND=* not determined.

**Table 3.** Some physicochemical qualities of the raw wastewater and treated final effluent.

Parameter	Raw wastewater		Treated effluent		Recommended target limits
	Range	Mean±SD	Range	Mean±SD	
pH	4.97–7.75	7.1 ± 0.44	6.7–7.7	7.1 ± 0.28	6–9^a^
Temperature (° C)	18–26	23 ± 2.3	18–26	22 ± 2.45	≤ 25 a
Turbidity (NTU)	86–1,000	573 ± 369	2.16–16	6.09 ± 3.64	0–1 a; ≤ 5^b^
TDS (mg/l)	311–907	452 ± 153	289–743	398 ± 110	0–450 a
DO (mg/l)	0.14–7.32	1.76 ± 1.78	2.38–6.78	4.46 ± 0.94	≥ 5^c^
COD (mg/l)	40–2,404	489 ± 701	4–960	143 ± 271	30^d^
NO_3_ (mg/l)	0.026–5.1	3.17 ± 1.32	0.25–6.95	4.56 ± 2.53	6^a^; 1–5^d^
NO_2_ (mg/l)	0.07–3.5	0.53 ± 0.93	0.07–6.95	0.88 ± 1.84	0–6^a^; <0.5^e^
PO_4_ (mg/l)	1.33–5.91	3.78 ± 1.26	0.05–0.73	0.34 ± 0.16	0.005^e^

Legend:

a Target limit for domestic water uses in South Africa [30];

b Target limit for effluent to be discharged into surface waters [31];

c Target limit for the support of aquatic life [32];

d Target limit for effluent to be discharged into the environment [33];

e Target limit that would reduce eutrophication in aquatic ecosystems [34].

**Table 4.** *In vitro* antibiotic susceptibility profile of the *Listeria* strains isolated from the effluents.

**Antibiotics**	**Number of isolates (%)**		
	Susceptible	Intermediate	Resistant
Amikacin (30 μg)	23(100)	0(0)	0(0)
Gentamycin(10 μg)	19(83)	0(0)	4(17)
Streptomycin(25 μg)	(15)65	0(0)	8(35)
Chloramphenicol(30 μg)	20(87)	0(0)	3(13)
Tetracyclin(30 μg)	19(83)	0(0)	4(17)
Ciprofloxacin(5 μg)	21(91)	1(4.5)	1(4.5)
Gatifloxacin(5 μg)	19(83)	2(8.5)	2(8.5)
Moxifloxacin(5 μg)	17(74)	3(13)	3(13)
Imipenem(10 μg)	19(83)	0(0)	4(17)
Meropenem(10 μg)	23(100)	0(0)	0(0)
Ertapenem(10 μg)	23(100)	0(0)	0(0)
Ampicillin(30 μg)	3(13)	0(0)	20(87)
Penicillin G(10 μg)	1(4.5)	1(4.5)	21(91)
Linezolid(30 μg)	18(78)	0(0)	5(22)
Aztreonam(30 μg)	21(91)	0(0)	2(9)
Erythromycin(15 μg)	4(17)	0(0)	19(83)
Cephalothin(30 μg)	17(74)	1(4)	5(22)
Ceftriaxone(30 μg)	21(91)	1(4.5)	1(4.5)
Sulphamethoxazole (25 μg)	8(35)	0(0)	15(65)
Trimethoprim(5 μg)	17(74)	0(0)	6(26)

**Table 5.** Multiple antibiotic resistances of *Listeria* strains isolated from the chlorinated effluents.

**Antibiotics**	**Number of isolates involved**	**Percentage (%)**
E, SMX, LZD, PG, AP	7^a^	31
E, LZD, PG, AP	2^b^	8.7
KF, E, SMX, LZD, PG, AP	2^b^	8.7
E, TM, LZD, MFX, PG, AP	1^b^	4.3
E, LZD, MFX, PG, AP	1^b^	4.3
C, KF, E, S, T, SMX, LZD, GAT, PG, AP	1^b^	4.3
E, S, T, SMX, LZD, MFX, PG, AP	1^b^	4.3
KF, E, S, SMX, TM, LZD, PG, AP	1^b^	4.3
CRO, KF, E, S, SMX, LZD, PG, AP,	1^b^	4.3
E, S, SMX, LZD, PG	1^b^	4.3
C, E, GM, S, SMX, TM, IMI, PG	1^b^	4.3
GM, TM, IMI, AP	1^b^	4.3
ATM, C, GM, S, T, TM, CIP, IMI, PG, AP	1^b^	4.3
GM, S, T, TM, LZD, IMI, PG, AP	1^b^	4.3

Total	22	95.7

Legend: ATM = Aztreonam; E = Erythromycin; AP = Ampicillin; LZD = Linezolid; PG = Penicillin G; KF = Cephalothin; SMX = Sulphamethoxazole; TM = Trimethoprim; MFX = Moxifloxacin; C = Chloramphenicol; S = Streptomycin; GAT = Gatifloxacin; CRO = Ceftriaxone; IMI = Imipenem; GM = Gentamycin; T = Tetracycline; CIP = Ciprofloxacin.

a One strain of *L. innocua* and six strains of *L. ivanovii*;

b Strains of *L. Ivanovii*.

**Table 6.** Occurrence of antimicrobial resistance genes in *Listeria* strains isolated from the final effluents.

Antibiotic resistance gene markers	Proportion of *Listeria* pathogens carrying the resistance genes
*penA*	0(0)
*ampC*	0(0)
*ermB*	0(0)
*ereA*	0(0)
*ereB*	0(0)
*su1I*	0(0)
*su1II*	5(22%)
